# GDNF Selectively Induces Microglial Activation and Neuronal Survival in CA1/CA3 Hippocampal Regions Exposed to NMDA Insult through Ret/ERK Signalling

**DOI:** 10.1371/journal.pone.0006486

**Published:** 2009-08-03

**Authors:** Francesca Boscia, Carla Lucia Esposito, Antonella Di Crisci, Vittorio de Franciscis, Lucio Annunziato, Laura Cerchia

**Affiliations:** 1 Dipartimento di Neuroscienze, Sezione di Farmacologia, Facoltà di Medicina e Chirurgia, Università degli Studi di Napoli “Federico II”, Naples, Italy; 2 Istituto per l'Endocrinologia e l'Oncologia Sperimentale del CNR “G. Salvatore”, Naples, Italy; Tel Aviv University, Israel

## Abstract

The glial cell line-derived neurotrophic factor (GDNF) is a potent survival factor for several neuronal populations in different brain regions, including the hippocampus. However, no information is available on the: (1) hippocampal subregions involved in the GDNF-neuroprotective actions upon excitotoxicity, (2) identity of GDNF-responsive hippocampal cells, (3) transduction pathways involved in the GDNF-mediated neuroprotection in the hippocampus. We addressed these questions in organotypic hippocampal slices exposed to GDNF in presence of N-methyl-D-aspartate (NMDA) by immunoblotting, immunohistochemistry, and confocal analysis. In hippocampal slices GDNF acts through the activation of the tyrosine kinase receptor, Ret, without involving the NCAM-mediated pathway. Both Ret and ERK phosphorylation mainly occurred in the CA3 region where the two activated proteins co-localized. GDNF protected in a greater extent CA3 rather than CA1 following NMDA exposure. This neuroprotective effect targeted preferentially neurons, as assessed by NeuN staining. GDNF neuroprotection was associated with a significant increase of Ret phosphorylation in both CA3 and CA1. Interestingly, confocal images revealed that upon NMDA exposure, Ret activation occurred in microglial cells in the CA3 and CA1 following GDNF exposure. Collectively, this study shows that CA3 and CA1 hippocampal regions are highly responsive to GDNF-induced Ret activation and neuroprotection, and suggest that, upon excitotoxicity, such neuroprotection involves a GDNF modulation of microglial cell activity.

## Introduction

Considerable interest has been devoted to neurotrophins as candidate neuroprotective agents for several neurodegenerative disorders since they promote neuronal survival, neuritic growth, and differentiation of several, but selective, neuronal populations. One such candidate is glial cell line-derived neurotrophic factor (GDNF) given its spectrum of demonstrated activities which includes, but is not limited to, potent trophic actions on a wide variety of neuronal populations of the central and peripheral nervous systems [1]. GDNF belongs to the GDNF family of ligands which consist of four structurally related neurotrophic factors - GDNF, neurturin, artemin , persephin- that signal through a multicomponent receptor composed of the transmembrane receptor tyrosine kinase Ret (rearranged during transfection) and high affinity glycosylphosphatidylinositol (GPI)-anchored proteins, the GDNF family α receptors 1–4 (GFRα1–4). Despite a cross-talk between the different ligands–GFRαs pairs, a preferred coreceptor molecule exists for each ligand, GDNF being the preferred high-affinity ligand for GFRα1 [1], [2].

*In vitro* studies have shown that following GDNF binding to GFRα1 the resulting complex recruits Ret, leading to its activation by dimerization and autophosphorylation at specific cytoplasmic tyrosine residues, thus initiating a number of downstream intracellular pathways [3]. On the other hand, a Ret-independent pathway of GDNF signalling that involves the association of GFRα-1 with the p140^NCAM^ isoform of the neural cell adhesion molecule (NCAM) and subsequent activation of Fyn and FAK kinases, has been as well demonstrated to take place in primary glial cells and neurons [4], [5].

In the last years a large number of studies demonstrated that GDNF provides potent neuroprotection in animal models of Parkinson's disease [6], motor neuron degeneration [7], [8], cerebral ischemia [9], and limbic seizure [10]. More importantly, the clinical use of GDNF for the treatment of the Parkinson's disease in humans is currently under evaluation [11]. The neuroprotective role of GDNF in the above mentioned neurodegenerative diseases lies on the widely recognized and potent pro-survival action on midbrain dopaminergic neurons [12], [13]; spinal cord motoneurons [7], [14], [15], noradrenergic neurons of the locus coeruleus [16], cerebellar Purkinje cells [17], cholinergic neurons of the basal forebrain [18], as well as peripheral sensory and autonomic neurons [19]. Interestingly, GDNF may exert trophic actions also in the hippocampus [20], a limbic region that is crucially involved in learning and memory processes. In addition, this region exhibits the distinctive feature of having neuronal populations which display differential vulnerability to several neurodegenerative stimuli [21]. Data obtained from *in vivo* studies indicated that GDNF and its receptors are widely expressed in the rat hippocampus [22]–[24]; furthermore, stroke, traumatic brain injury, or kainate-induces seizures significantly increases GDNF and their receptors mRNA expression in this limbic region [10], [25]. *In vitro*, GDNF has been found to protect hippocampal slice cultures and primary cortical neurons against toxic activation of the N-methyl-D-aspartate (NMDA) receptor [26], [27], where its application reduced the NMDA-induced calcium influx [27]. Although these studies suggest an important role for this trophic factor in this region following excitotoxic insults, the signalling pathways involved in GDNF-mediated neuroprotection in each hippocampal subfield and the identity of hippocampal cells that respond to GDNF stimulation still remains elusive.

In the present study, by combining Western blotting, immunofluorescence and confocal analysis on rat organotypic hippocampal slice cultures (OHSCs), we first investigated the intracellular signalling mechanisms triggered by acute or chronic GDNF exposure in the different subfields of the hippocampus. Then, we used the NMDA-induced neurotoxicity in OHSCs as experimental model to insight the neuroprotective actions of GDNF in the different hippocampal subregions. Finally, we explored the identity of hippocampal cells that respond to GDNF stimulation following NMDA-induced neurodegeneration.

## Results

### GDNF stimulation activates Ret/ERK signalling in rat organotypic hippocampal slices

**Figure 1:**
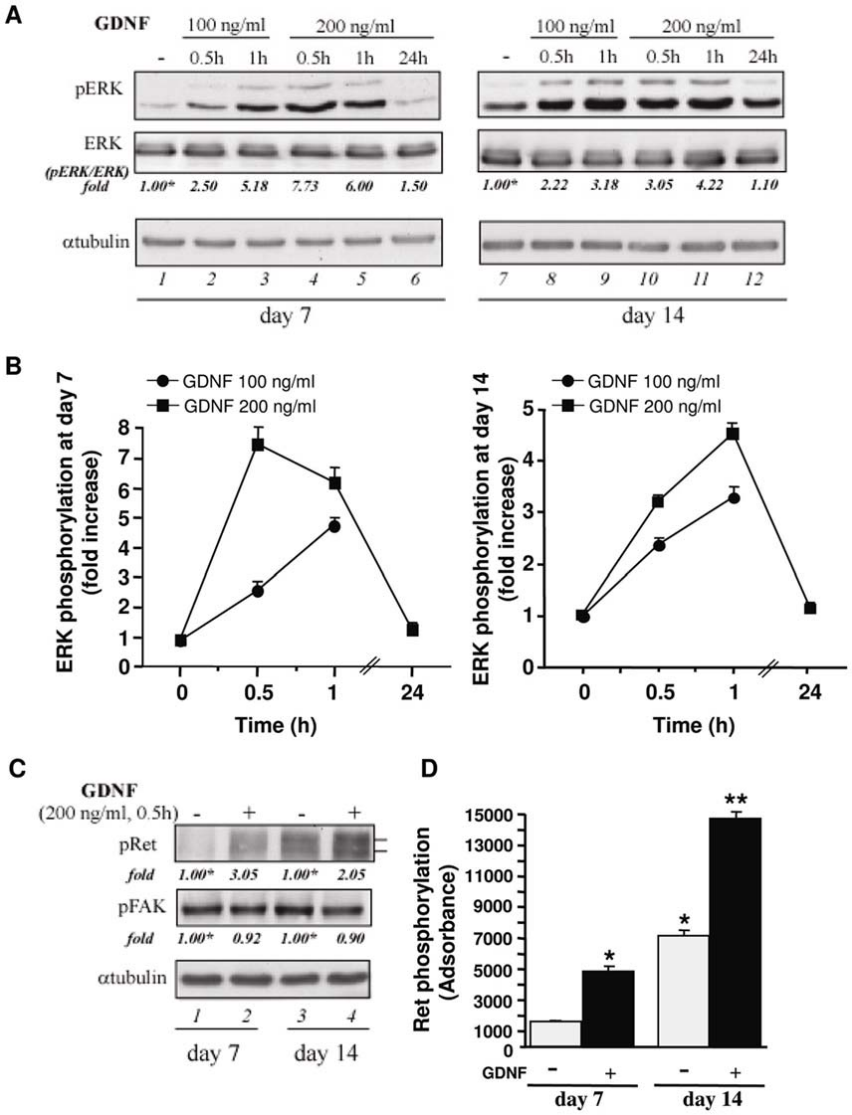
GDNF activates Ret/ERK signalling in rat OHSCs. (A) Slice cultures at DIV 7 (left) and 14 (right) following dissection were left untreated (lanes 1 and 7) or stimulated with GDNF (100 ng/ml and 200 ng/ml) for the indicated incubation times. Crude extracts were immunoblotted with anti-pERK antibodies and then the filters were stripped and reprobed with anti-ERK antibodies. To confirm equal loading the filters were probed with anti-αtubulin antibodies. Quantitations are done on the sum of the two ERK-specific enhanced chemiluminescence bands of 44 and 42 kDa. Intensity of bands have been calculated using the NIH Image Program on at least two different expositions to assure the linearity of each acquisition. Fold values are expressed relative to the reference points (lanes 1 and 7), arbitrarily set to 1 (labelled with asterisk). (B) Plots of fold values corresponding to the induction of pERK normalized for the amount of ERK in each lane of immunoblotting reported in A. (C) Slice cultures at DIV 7 (left) and 14 (right) following dissection were left untreated or stimunlated with 200 ng/ml for 30 min. Crude extracts were immunoblotted with anti-pRet and anti-pFAK antibodies. To confirm equal loading the filters were stripped and reprobed with anti-αtubulin antibodies. Quantitations are done on the sum of the two Ret-specific enhanced chemiluminescence bands of 170 and 150 kDa corresponding to different glycosylation states of Ret. Fold values are expressed relative to controls (lanes 1 and 3), arbitrarily set to 1 (see A). (D) Plots of absorbance corresponding to the results reported in C for Ret phosphorylation in the absence of GDNF (grey column), or in the presence of GDNF (black column), for both DIV 7 and 14. * p<0.05 versus control group at 7DIV. ** p<0.05 versus all groups.

With the aim to investigate the intracellular signalling mechanisms triggered by GDNF stimulation of rat organotypic hippocampal slices, we first tested the effect on ERK phosphorylation in slice cultures at day of culture *in vitro* (DIV) 2, 7, and 14 following dissection. As shown in Fig. 1A and B, application of GDNF under serum-free medium increased the phosphorylated ERK levels with respect to the unstimulated slices, in a dose- and time-dependent manner. Both concentrations of 100 ng/ml or 200 ng/ml of ligand were effective in causing a strong activation of ERK at 30 minutes that was sustained up to 60 minutes. A return to basal levels of ERK phosphorylation was observed following 24 h of GDNF application. Comparable results were obtained for cultures from 2 (data not shown), 7 (Fig. 1A and B, left) and 14 DIV (Fig. 1A and B, right). In order to determine whether the stimulation of phosphorylated ERK levels by GDNF treatment was mediated by a Ret-dependent or by a p140^NCAM^-dependent mechanism, we stimulated hippocampal slices at 7 and 14 DIV with 200 ng/ml GDNF for 30 minutes and looked for Ret and FAK tyrosine phosphorylation, the latter being an upstream effector of NCAM-dependent GDNF signalling [4]. As shown, following GDNF stimulation, a marked increase in Ret phosphorylation with respect to the basal levels was clearly observed (Fig. 1C, upper panel, compare lanes 2 and 4 to lanes 1 and 3, respectively and Fig. 1D), whilst no effect on FAK phosphorylation was detectable (Fig 1C, middle panel), thus indicating that in OHSCs GDNF signals mainly through Ret rather than through the p140^NCAM^-dependent, Ret-independent, pathway. Since both the basal and GDNF-induced levels of Ret phosphorylation (Fig. 1C, compare lane 3 to 1, and Fig. 1D) were higher in the unstimulated slices from 14 DIV with respect to those from 7 DIV, mature cultures at 14 DIV were used to perform the next experiments.

### GDNF activates Ret/ERK signalling in a specific population of IB4-positive cells in the CA3 subregion

**Figure 2:**
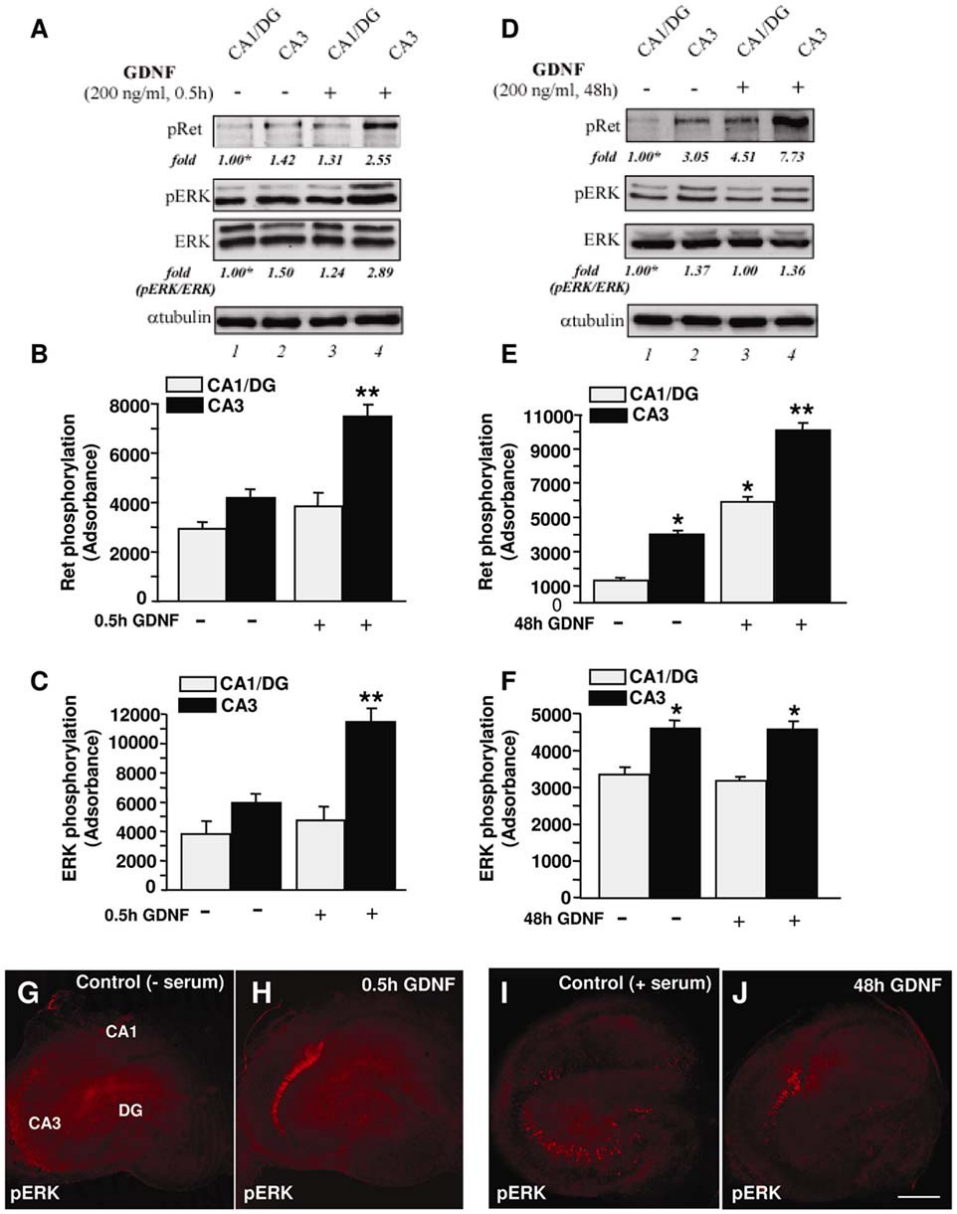
Acute or chronic GDNF treatment activates Ret/ERK signalling preferentially in the CA3 region of OHSCs. Rat OHSCs (14 DIV) were left unstimulated or treated with 200 ng/ml GDNF for 30 min (A) or 48 h (D); the CA1 *plus* DG regions were separated from the CA3 region and the crude extracts were immunoblotted with anti-pRet or anti-pERK antibodies. Then the filters were stripped and reprobed with anti-ERK antibodies. To confirm equal loading the filters were probed with anti-αtubulin antibodies. Quantitations were done as reported in legend to Figure 1A and relative abundances are expressed relative to CA1/DG regions (in the absence of GDNF stimulation), arbitrarily set to 1 (labelled with asterisk, lanes 1). (B) and (E) Plots of absorbance corresponding to the results reported in A and D, respectively, for Ret phosphorylation (upper panel) and ERK phosphorylation (lower panel). * p<0.05 versus CA1/DG control group. ** p<0.05 versus all groups (G-J) Distribution of pERK immunoreactivity in control serum-free-exposed OHSCs for 30 minutes (G), in control serum-exposed OHSCs (I), in mature OHSCs exposed to acute (H) or to chronic -GDNF exposure (J). Scale bar: 400 µm in G-J

We thus determined the extent of Ret and ERK activation following GDNF stimulation throughout the hippocampal subfields. To this aim, we first exposed mature OHSCs to an acute or chronic GDNF stimulation; the CA1 *plus* DG regions were separated from the CA3 region and the crude extracts were prepared and analysed by immunoblotting with anti-pRet or anti-pERK antibodies. Following 30 minutes of GDNF treatment (acute stimulation) a clear increase of both Ret and ERK phosphorylation occurred essentially in the CA3 region (Fig. 2A, compare lanes 4 to 2, and Fig. 2B-C). In addition, in control serum-exposed slices the basal levels of Ret phosphorylation were higher in CA3 if compared to CA1/DG and further increased by a prolonged, chronic (48 h) GDNF treatment (Fig. 2D, compare lane 4 to 2 and Fig. 2E-F).

As shown in Figure 2G-J, we further confirmed by immunofluorescence analyses in HOSCs that the CA3 is the hippocampal region that is more responsive to the action of GDNF. In fact, acute GDNF stimulation induced a dense phoshorylated ERK labelling specifically localized in the pyramidal layer of CA3 subfield (Fig. 2G-H). Although less intense and more diffuse, a similar pattern of regional ERK phosphorylation in the CA3 subfield was observed under GDNF chronic stimulation in the presence of serum (Fig. 2I-J).

**Figure 3:**
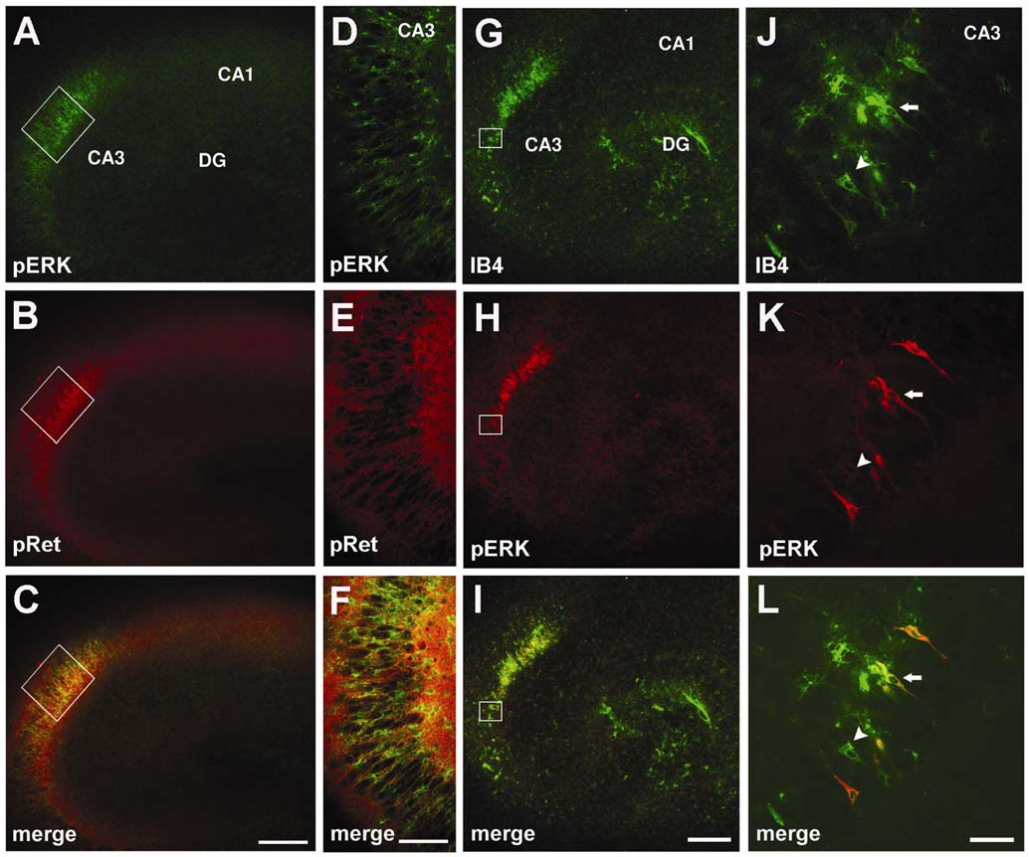
Co-localization of pERK/pRet and of pERK/IB4 immunoreactivity in the CA3 region following acute GDNF exposure. (A-C): Low magnification fluorescence microphotographs of OHSCs immunolabeled for pERK (green), and pRet (red) in the CA3 region. (D-F) Higher magnification fluorescence images of the frame depicted in A-C displaying both pERK (green) and pRet (red) labelling in strata pyramidale of CA3 region. (G-I): Low magnification fluorescence microphotographs of OHSCs immunolabeled for IB4 (green), and pERK (red) in the CA3 region. (J-L): Higher magnification of frames depicted in G-I displaying cells double-labelled for both pERK and IB4 signals (arrows) and a cells positive for pERK only (arrowheads). Scale bars: 200 µm in A-C and G-I; 100 µm inD-F, 50 µm in J-L.

Double immunofluorescence labelling with anti-pERK and anti-pRet antibodies revealed the co-existence of both phosphorylated Ret and pERK immunosignal of CA3 region of HOSCs following acute GDNF stimulation (Fig. 3A-C). A large number of cells in the pyramidal layer were double-labelled for both phosphorylated ERK and Ret proteins (Fig. 3D-F).

In order to characterize the hippocampal cell population that is responsive to GDNF we determined whether that population might also be positive for IB4 labelling, a lectin that has been recently used to identify a subpopulation of GDNF-responsive neurons [28]. Co-localization experiments revealed that most of the cells positive for pERK after GDNF stimulation in the CA3 region were double-labelled for IB4 (Fig. 3G-L). No co-localization of pERK-positive cells with NeuN, GFAP, OX-42, Olig-2 or NF-200 markers, which identify mature neurons, astrocytes, microglia cell types, oligodendrocyte precursors and neurofilament proteins of neuronal cells, respectively, was observed (data not shown). These results, taken together, indicate that GDNF may induce Ret activation and the consequent downstream signalling in a specific population of hippocampal CA3 cells in the pyramidal layer that are IB4-positive.

### Neuroprotective effects of GDNF against NMDA-induced neurodegeneration in rat organotypic hippocampal slices

**Figure 4:**
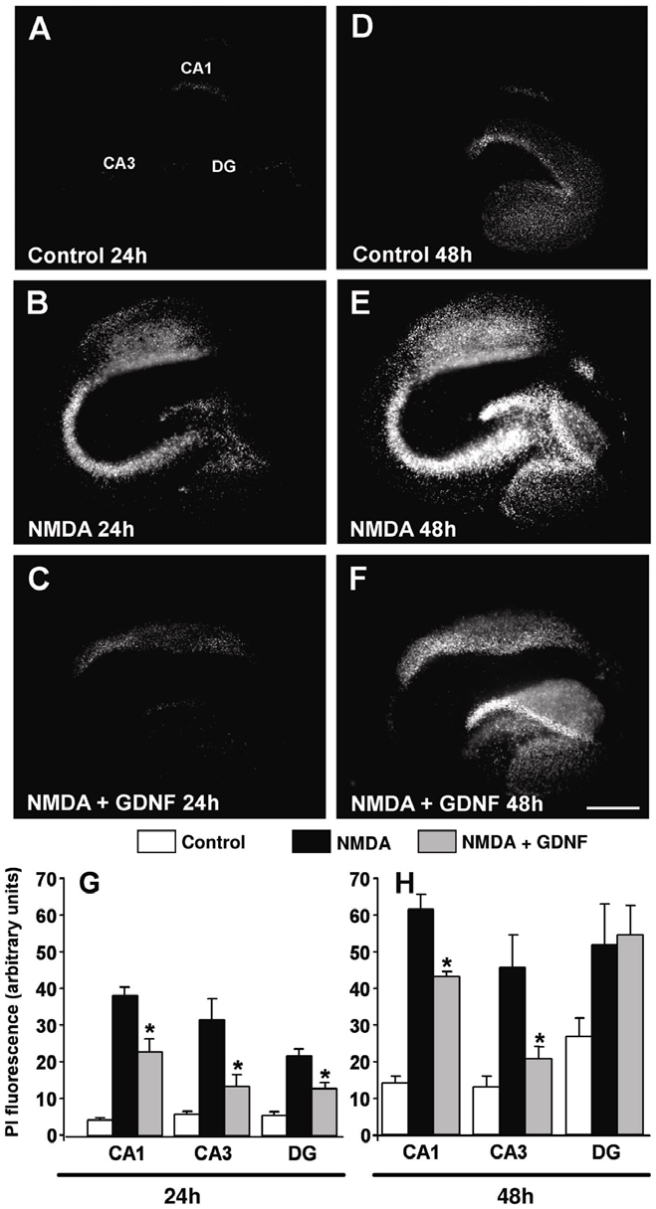
Effect of GDNF during NMDA-induced neuro toxicity in rat OHSCs. (A-C): PI fluorescence staining patterns observed in representative OHSCs 24 h following their exposure to the experimental conditions indicated in each panel. (D-F): PI fluorescence staining patterns observed in representative OHSCs 48 h following their exposure to the experimental conditions indicated in each panel. Scale bar: 400 µm in A-F. (G-H): quantification of cell damage (densitometric analysis of PI fluorescence) was performed in selected hippocampal subregions (CA1, CA3, DG). Data are expressed as arbitrary units (A.U.) of PI fluorescence intensity. GDNF was used at the concentration of 200 ng/ml. Each data point is the Mean±S.E.M. of the data obtained in 20–24 OHSCs from 3 separate experiments. Asterisks denote values statistically different from those obtained in the respective NMDA-treated OHSCs (p<0.05).

Given that the CA3 is the hippocampal subregion most responsive to GDNF receptor activation, we determined whether the neuroprotective action of GDNF was mainly exerted in this region. As previously reported, exposure of rat OHSCs to 10 µM NMDA resulted in a time-dependent enhancement of propidium iodide (PI) uptake, that was detectable after at least 4–8 h of NMDA exposure and was further increased after 24 and 48 h [21]. When OHSCs were exposed for 48 h to 10 µM NMDA, cell death preferentially occurred in the CA1 pyramidal cell layer and, to a lesser degree, in the CA3 and DG hippocampal subfields (Fig. 4).

A marked neuroprotective effect was observed when GDNF was added to the incubation medium 48 h before NMDA exposure (Fig. 4). Shorter incubation times of GDNF were not sufficient to exert such neuroprotective effects (data not shown). Cell death was significantly reduced in all hippocampal subfields 24 h after NMDA exposure (Fig. 4 panels A-C and G), whereas after 48 h of treatment the CA1 and CA3 regions were the most sensitive regions to GDNF induced neuroprotection (Fig. 4 panels D-F and H).

**Figure 5:**
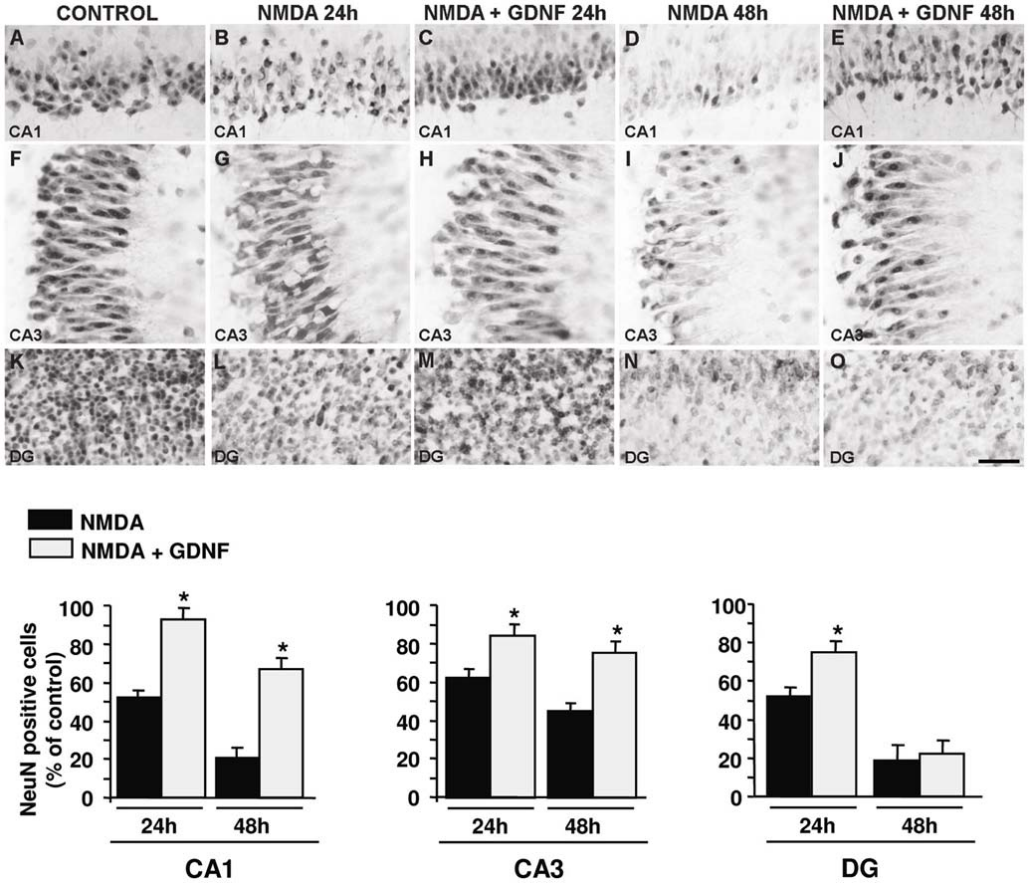
GDNF neuroprotective effect on NMDA-induced cell death revealed by NeuN immunohistochemistry. A-E: NeuN immunoreactivity in the CA1 pyramidal layer of control OHSC (A); NMDA-exposed HOSC for 24 h in absence (B) or in presence of GDNF (C); and of NMDA-exposed HOSC for 48 h in absence (D) or in presence of GDNF (E). F-J: NeuN immunoreactivity in the CA3 pyramidal layer of control OHSC (F); NMDA-exposed HOSC for 24 h in absence (G) or in presence of GDNF (H); and of NMDA-exposed HOSC for 48 h in absence (I) or in presence of GDNF (J). K-O: NeuN immunoreactivity in the granule cell layer of dentate gyrus (DG) of control OHSC (K); NMDA-exposed HOSC for 24 h in absence (L) or in presence of GDNF (M); and of NMDA-exposed HOSC for 48 h in absence (N) or in presence of GDNF (O). Scale bar: 50 µm in A-O. Quantification of NeuN-positive cells in the CA1, CA3 and DG is reported. In each experimental group cells were counted in 9 non-overlapping microscope fields subfields from three different slices. Data are expressed as percentage of NeuN-positive cells counted in control slices. *p<0.05 versus NMDA-exposed HOSCs.

To verify whether GDNF prevention of NMDA-induced PI uptake involved neuronal cell protection, immunohystochemical analysis for the neuron-specific nuclear protein NeuN was performed. Organotypic hippocampal slices exposed for 24 and 48 h to NMDA showed an evident loss of NeuN staining intensity in CA1 and CA3 neurons (Fig. 5), that was rescued by GDNF treatment. Interestingly, NeuN staining in the CA1 and, particularly in the CA3 subfield of GDNF-treated OHSCs exposed to NMDA was similar to controls (Fig. 5F-J), thus reinforcing the finding that GDNF exerted a strong protective action on CA3 and CA1 neurons.

### Ret receptor is activated in IB4-positive microglial cells of hippocampal organotypic cultures pretreated with GDNF and exposed to NMDA

**Figure 6:**
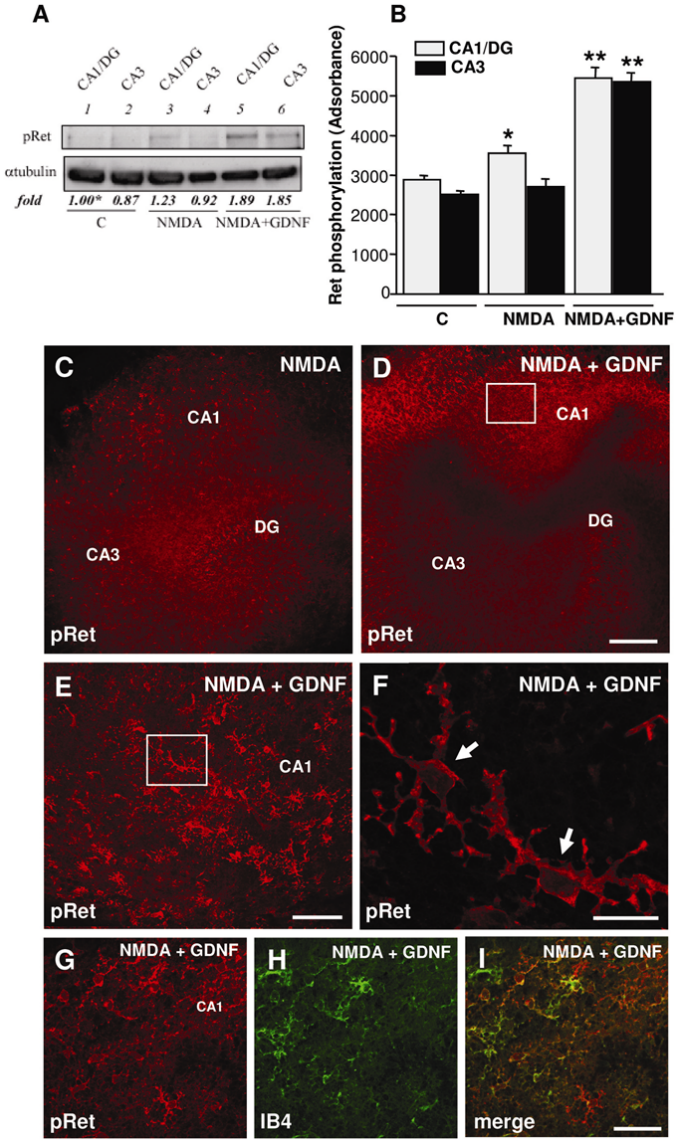
GDNF-dependent Ret activation in OHSCs following NMDA exposure. (A) OHSCs were left untreated (lanes 1–2) or exposed to NMDA in the absence (lanes 3–4) or in the presence (lanes 5–6) of GDNF for 24 h. Crude extracts were prepared from the CA1 *plus* DG regions and from the CA3 region immunoblotted with anti-pRet antibodies. To confirm equal loading the filters were probed with anti-αtubulin antibodies. Quantitations were done as reported in legend to Figure 1A and relative abundances are expressed relative to CA1/DG control regions, arbitrarily set to 1 (labelled with asterisk, lanes 1). (B) Plots of absorbance corresponding to the results reported in A for Ret phosphorylation. * p<0.05 versus control and NMDA groups. ** p<0.05 versus all groups. C-I: Distribution of pRet immunoreactivity in mature OHSCs exposed to NMDA-induced neurodegeneration in absence (C) or in presence of GDNF exposure (D). (E) Higher magnification of the frame depicted in D displaying intensely stained pRet- positive cells scattered troughout the CA1 region. (F) Higher magnification of the frame depicted in E, displaying two cells (arrows) with microglial morphology intensely labelled by the pRet antibody. G-I: Confocal microscopic images of OHSCs depicting microglial cells displaying both pRet (red) and IB4 (green) immunoreactivity (thick arrows) in the CA1 region following NMDA plus GDNF exposure. Scale bars: 200 mm in C-D; 100 mm in E; 20 mm in F, 50 mm in G-I.

Given the widespread observed neuroprotective actions of GDNF against NMDA-induced neurodegeneration even in the subregions that poorly respond to GDNF stimulation, (cfr Fig. 3), we examined by immunoblotting the activation pattern of the Ret receptor following NMDA insult in presence or in absence of GDNF (Fig. 6A-B). To this aim, rat OHSCs were left untreated (lanes 1–2) or exposed to NMDA in the absence (lanes 3–4) or in the presence (lanes 5–6) of GDNF. When HOSCs were exposed to NMDA for 24 h, Ret activation was observed in CA1/DG subfields but not in the CA3 subregion (compare lane 3 to lane 1). Twenty-four hours GDNF exposure of NMDA-exposed slices, stimulated Ret phosphorylation in the CA3 region (compare lane 6 to lane 4 and 2) and further increased phosphorylated Ret protein levels in CA1/DG subfield (compare lane 5 to lanes 1 and 3). In accordance with biochemical studies, immunofluorescence staining performed with the anti-pRet antibody in the CA1 subregion revealed that pRet immunoreactivity increased in presence of NMDA and was further pronounced in NMDA plus GDNF-exposed slices. Interestingly, the anti-pRet antibody intensely stained small and scattered cells throughout the slices which resemble microglia cell populations. Consistently, double immunolabelling experiments performed with pRet and both IB4, or OX-42 (data not shown) microglial markers confirmed the presence of pRet receptors on microglial cells either following NMDA (data not shown) and NMDA plus GDNF exposed slices (Fig. 6G-I). By contrast, pRet immunostaing was not evident in microglia under control conditions (data not shown).

## Discussion

In the present study, by using rat hippocampal organotypic slices subjected to NMDA-induced neurotoxicity as model system, we investigated the neuroprotective role of GDNF in the hippocampus.

### Rat organotypic hippocampal slices express functional GDNF receptor complex

First, we demonstrated that GDNF stimulation induces activation of Ret tyrosine kinase receptor and its downstream effector ERK in a dose and time dependent manner. By combining immunoblotting and immunofluorescence experiments performed on separated CA1, CA3 or DG subregions, we established that a GDNF treatment either if rapid (30 min) or prolonged (48 h) induces a strong activation of the Ret/ERK signalling preferentially in the CA3 subfield. Recently, NCAM has been identified as an alternative signalling receptor for GDNF [5]. Indeed, it has been demonstrated that GDNF stimulates Schwann cell migration and axonal growth in hippocampal and cortical neurons via binding to p140^NCAM^ isoform in the presence of GFRα, but independently of Ret, thus leading to activation of the cytoplasmic protein tyrosine kinases Fyn and FAK, two signaling mediator downstream of p140^NCAM^ [4]. In our experimental conditions, GDNF treatment of OHSC did not activate FAK, thus indicating that in OHSC GDNF doesn't, or poorly, signals through the p140^NCAM^-dependent pathway.

In line with our results, in situ hybridization and immunohystochemical experiments demonstrated that both Ret mRNA and protein are much more abundant in CA2 and CA3 pyramidal sector of rat postnatal [24], and adult human brain [29]. Despite the GDNF-dependent activation of Ret was not addressed by these studies, they suggest the involvement of Ret and GFRα receptors signalling in processes fundamental for both the functional activity and maintenance of the mature hippocampal neurons and the organization of this cortical region during development.

Furthermore, with the attempt to elucidate the identity of hippocampal cells that respond to GDNF stimulation, we found activated both Ret and ERK proteins in a subpopulation of cells of the pyramidal layer that by immunofluorescence co-localization experiments with specific markers, NeuN, GFAP, OX-42, Olig-2 and NF-200 do not resemble neither mature neurons, astrocytes, microglia, oligondendrocyte precursors nor neuronal cells. Intriguingly, these cells double-labelled for both phosphorylated ERK and Ret proteins, resulted positive for the staining with IB4, a lectin that although largely used as a marker for microglia [30], has been recently reported to identify a subpopulation of GDNF-responsive nociceptive dorsal root ganglion Ret-positive neurons [28], [31], [32].

### GDNF protects CA3 and CA1 region against NMDA-induced neurodegeneration

We found that GDNF displayed a broad neurotrophic action following NMDA treatment that resulted in protecting, even if at different degree, CA1 and the less vulnerable CA3 subfields. Interestingly, we found that, while in the absence of the excitotoxicity insult, GDNF stimulates Ret-dependent signalling mainly in the subpopulation of IB4-positive cells of the CA3 region (see above), the pattern of GDNF-dependent activation of Ret changes following NMDA treatment. Indeed, we observed that a pre-treatment of hippocampal slices with GDNF before inducing the insult, caused a pronounced increase of Ret phosphorylation in both CA1 and CA3 region. More specifically, we observed an enhanced activation of Ret in IB4-positive microglial cells in the CA1, and with a lesser degree in the CA3 region. In accordance with our findings, it has been demonstrated that the GDNF receptors, Ret and GFRα1, are expressed in rat primary cultured microglia [33], [34] and that exogenous GDNF administration may not only have a protective effect on neurons, but may also have a modulatory role in microglial activities including survival and proliferation [33], [35]. In fact, though microglial cells have been reported to act as scavenger cells or mediator of inflammatory responses in several neurodegenerative states, several studies support a trophic role for these cells in tissue repair and remodelling by secreting cytokines and/or growth factors [36]. Consistently with these observations, it can be hypothesized that treating hippocampal slices with GDNF during the insult might cause a regulation of microglial activities trough Ret activation, thus providing trophic support in the most damaged CA1 region.

Collectively, this study shows that CA3 and CA1 hippocampal regions are highly responsive to GDNF-induced Ret receptor activation and neuroprotection, and suggest that, upon excitotoxicity, such neuroprotective action involves a GDNF modulation of microglial cell activity. Our results open new insights on the mechanisms involved in the neuroprotective actions of GDNF.

## Materials and Methods

### Drugs and materials

Propidium iodide was from Molecular Probes Europe BV (Leiden, The Netherlands). Recombinant GDNF was from Alomone Labs (Jerusalem, Israel). All media and sera for OHSCs were purchased from Gibco (Milan, Italy). NMDA was dissolved directly in serum-free medium (stock solutions of 10 mM) and then diluted to the desired concentration; this stock solutions was stored at 4°C for 1 month. Stock solutions were diluted to achieve the desired drug concentration immediately before use.

### Rat organotypic hippocampal slice cultures

OHSCs were prepared as previously described in detail [21]. Briefly, 400 µm thick transverse hippocampal slices were prepared from 7–9 days old Wistar rat pups (Charles River, Calco, Italy) using a McIlwain tissue chopper (Campden Instruments, Leicester, UK) in a sterile environment and placed into ice-cold HanKs' balanced salt solution (HBSS, Gibco-BRL, Renfrewshire, UK) supplemented with 5 mg/ml glucose and 1% (v/v) Fungizone (Amphotericin B). Cultures were then transferred to humidified semiporous membrane (30 mm Millicell-CM tissue culture plate inserts of 0.4 µm pore size from Millipore, Rome, Italy) in 6-well tissue culture plates (5 slices per membrane). Each well contained 1.2 ml of tissue culture medium consisting of 50% minimal essential medium (MEM), 25% HBSS, 25% heat-inactivated horse serum, 5 mg/ml glucose, 1 mM glutamine and 1% Fungizone; the medium was replaced twice a week. OHSCs were mantained at 37°C, 100% humidity, in a 95% air/5% CO_2_ atmosphere. Experiments and animal use procedures were in accordance with the National Institutes of Health guide for the care and use of Laboratory animals (NIH Publications No. 8023, revised 1978); the experimental protocol was approved by the Animal Care Committee of the “Federico II” University of Naples.

### Stimulation of OHSCs with GDNF

Cultures were exposed to GDNF in two ways. To examine the acute effects of GDNF treatments in OHSCs we used the “drop application”, in which a 5 µl drop of 100–200 ng/ml GDNF solution was applied to the top of each slice, as described by Marty et al. [37]. This procedure has been show to accelerate the penetration of neurotrophins [38]. Control slices received a drop of the vehicle alone. To examine the chronic effects of GDNF treatments in OHSCs, 200 ng/ml GDNF was included in the culture medium beneath the membrane insert. The cultures were exposed to GDNF for 48 h before inducing the neurotoxic insults.

### NMDA exposure

NMDA exposure was performed in OHSCs as previously described [21]. OHSCs were removed from normal serum-containing medium (NM), washed in serum-free medium (SFM, consisting in NM with serum replaced with MEM) and exposed to 10 µM NMDA (with or without additional compounds) for 24–48 h in fresh SFM. Control OHSCs were kept in SFM.

### Assessment of cell death and image analysis

Cell injury was assessed using the fluorescent dye propidium iodide. PI is a very stable and highly polar compound which only enters cells with damaged or leaky plasmamembranes, binds to DNA, and emits a brightly red fluorescence when exposed to blue-green light. PI is non-toxic to neurons and has been used as a marker of neuronal membrane integrity and cell damage. Briefly, at the beginning of the experiment, 5 µg/ml PI was added to the culture medium for 30 minutes to check for slice viability; OHSCs in which significant PI fluorescence was detected were excluded from further studies. PI uptake was recorded by a digital camera (Media Cybernetics, Silver Springs, MD, USA) mounted on a Nikon Eclipse 400 fluorescence microscope (Nikon Instruments, Florence, Italy; excitation 510–560 nm, emission 590 nm). Preliminary tests were carried out on maximally fluorescent OHSCs to determine a combination of illumination intensity and integration time which would fully exploit the 12-bit dynamic range of the imaging system without saturating it. For densitometric measurements, the digital photos were analyzed with the Image Pro-Plus software (Media Cybernetics, Silver Springs, MD, USA), after freehand outlining of the CA1, CA3 and DG neuronal layers, as previously described [21].

### Western blotting experiments

In some experiments the slices were transferred to a glass slide at ice temperature, and the CA3 areas were dissected out from CA1/DG subregions under a stereo microscope and stored at ±80°C until assay. The slices were lysed in 50 mM Tris-HCl pH 8.0 buffer containing 150 mM NaCl, 1% Nonidet P-40, 2 µg/ml aprotinin, 1 µg/ml pepstatin, 2 µg/ml leupeptin, 1 mM Na_3_VO_4_. The solution was centrifuged at 16000 *g* for 30 min at 4°C and the residue was discarded. Protein concentration was determined by the Bradford assay using bovine serum albumin (BSA) as the standard and the crude extracts were subjected to 10% SDS-PAGE. Gels were electroblotted into polyvinylidene difluoride membranes (Millipore Co., Bedford, MA), and filters were probed with the indicated primary antibodies: anti-(Tyr1062-phosphorylated) Ret (indicated as pRet), anti-ERK1 (C-16) (Santa Cruz Biotechnology Inc, Santa Cruz CA); anti-(Ser473-phosphorylated), anti-phosphorylated-p44/42 mitogen-activated protein kinase E10 (indicated as pERK, Cell Signaling, Beverly, MA); anti-α-tubulin (DM 1A; Sigma, St. Louis, MO); anti- (Tyr397-phosphorylated) FAK (indicated as pFAK; Upstate, Lake Placid, NY). Proteins were visualized with peroxidase-conjugated secondary antibodies using the enhanced chemiluminescence system (Amersham-Pharmacia Biosciences LTD, Uppsala, Sweden). When indicated, membranes were stripped in 62.5 mM Tris-HCl pH 6.7, 0.1 M 2-mercaptoethanol, 2% SDS for 30 min at 55°C.

### Immunohystochemistry and immunofluorescence confocal microscopy

Immunohistochemistry in OHSCs was performed as described by Boscia et al. [39]. Briefly, OHSCs were washed in 0.1 M phosphate buffer at pH 7.4 (PB) and fixed in 4% w/v paraformaldehyde an 0.1 M PB for 1 h at room temperature (RT). Slices were washed three times in 50 mM Tris- buffered saline pH 7.4 (TBS) for 30 minutes between each step. Slices were first blocked in 3% (w/v) BSA and Triton X-100 0.25%, and then incubated with the following primary antibodies: rabbit polyclonal anti-pERK (1∶500; Cell Signaling, USA), mouse monoclonal anti-pERK (1∶1000, Cell Signaling, USA), rabbit polyclonal anti-pRet (Tyr1062) (1∶ 200; Santa Cruz Biotechnology Inc), mouse monoclonal anti-NeuN (1∶2000; Chemicon, Milan, Italy); Isolectin *Bandeiraea Simplicifolia* B4 (IB4) directly conjugated to FITC (FITC-IB4; 1∶ 200; Sigma, Italy) at 4°C.

After 48 h, the slices processed for light microscopy were first incubated in biotinylated horse anti-mouse or goat anti-rabbit IgG (each at 1∶200 dilution, Vector Laboratories, Burlingame, CA, USA) for 2 h and then in avidin–biotinylated horseradish peroxidase complex (Elite ABC, 1∶300 dilution, Vector) for 1.5 h, always at room temperature. The peroxidase reaction was developed using 3,3-diaminobenzidine 4-HCl (DAB) as a chromogen and 0.05% H_2_O_2_. After the final wash, sections were dehydrated, coverslipped, and processed for microscope analysis. Images were acquired by a digital camera (Coolsnap, Media Cybernetics, Silver Springs, MD, USA) mounted on a Nikon Eclipse 400 microscope.

Following 48 h of primary antibody incubation, the slices processed for single or double-labelling fluorescence experiments were incubated in single or in a mixtures of fluorescent-labelled secondary antibodies Alexa 488-conjugated anti-mouse IgG and Alexa 568-conjugated anti-rabbit IgG (each at 1∶200 dilutions; Molecular Probes, Eugene, OR) for 3 h at RT. Finally, the sections were washed in TBS, transferred onto microscope slides and covered with Vectashield mounting medium (Vector Laboratories, Inc., and California). Immunofluorescence images were observed using a Zeiss LSM 510 META Laser-Scanning Confocal Microscope (Carl Zeiss, Thornwood, NY, USA) by which single images were taken with an optical thickness of 0.7 µm and a resolution of 1,024×1,024 pixels. Controls of the methods in the present experiments included replacement of the primary antisera with normal serum (1∶200). To control for a possible cross-reactivity between IgGs in double immunolabeling experiments, some sections were processed through the same immunocytochemical sequence except that primary antisera were replaced with normal serum, or only one primary antibody was applied, but the full complement of secondary antibodies was maintained. In addition, the secondary antibodies utilized were highly pre-adsorbed to the IgGs of numerous species. Tissue labelling without primary antibodies was also tested to exclude autofluorescence. No specific staining was observed under these control conditions, thus confirming the specificity of the immunosignals.
